# COPB2 gene silencing inhibits colorectal cancer cell proliferation and induces apoptosis via the JNK/c-Jun signaling pathway

**DOI:** 10.1371/journal.pone.0240106

**Published:** 2020-11-19

**Authors:** Yan Wang, Guangmei Xie, Min Li, Juan Du, Min Wang

**Affiliations:** 1 Gansu Provincial Hospital, Lanzhou, Gansu, China; 2 Institute of Pathology, School of Basic Medical Sciences, Lanzhou University, Lanzhou, Gansu, China; 3 Gansu Provincial Maternity and Child-care Hospital, Lanzhou, Gansu, China; Chung Shan Medical University, TAIWAN

## Abstract

**Objectives:**

Colorectal cancer (CRC) is one of the most common malignant human tumors. It is associated with high morbidity and mortality rates. In recent years, tumor gene therapy has emerged as a promising new approach for colorectal cancer therapy. Herein, we identify and analyze the role of COPB2 (coatomer protein complex, subunit beta 2) in proliferation and apoptosis of CRC cells.

**Methods:**

To investigate the role of COPB2 in the proliferation and apoptosis of CRC cells, a shCOPB2 vector and a shCtrl vector were constructed for transfection into RKO and HCT116 cells. Cells proliferation was subsequently measured via cell counting kit-8 (CCK8) assay and Celigo cell counting assay. Apoptosis was measured via flow cytometry. The activity level of Caspase 3/7 was measured. Finally, the level of several JNK/c-Jun apoptosis pathway-related proteins were measured to characterize the mechanism of apoptosis.

**Results:**

Our results showed that the proliferation rate was decreased and the apoptosis rate was increased in shCOPB2-treated RKO and HCT116 cells compared to those in controls. After the silencing of COPB2, JNK/c-Jun signal pathway activation was increased, the expression levels of apoptosis pathway-related proteins, such as Bad, p53 and Caspase 3, were also increased.

**Conclusion:**

COPB2 gene silencing can inhibit RKO and HCT116 cells proliferation and induce apoptosis via the JNK/c-Jun signaling pathway.

## Introduction

CRC is the most common malignant tumor of the human digestive system [1]. Nearly 1.2 million new cases of CRC are diagnosed annually, and more than 600,000 death occur worldwide due to CRC every year [2,3]. CRC accounts for approximately 8% of all cancer deaths [4] and is the third-leading cause of death from cancer among adults around the world [5,6]. High incidence and mortality rates due to CRC have been identified in China. The incidence of the disease has marked geographical distribution characteristics [7,8]. The incidence in southeastern coastal cities is higher than that in northwest rural areas [8]. Treatment for CRC primarily based on surgery [9,10], but often includes chemotherapy [11], radiotherapy [12], and biotherapy, and other therapies [13,14]. With advancements in genetic engineering technology, gene therapy has attracted increasing attention as a novel method of CRC treatment [15–17].

COPB2 coatomer protein complex, subunit beta2 (COPB2) is primarily distributed in the endoplasmic reticulum and golgi apparatus, where it plays a significant role in transportation of various substances between the intracellular and intercellular environments [18–22]. COPB2 levels reportedly correlated with the occurrence and development of tumors [23], such as PC3 prostate cancer [24]. We have previously reported that COPB2 is highly expressed in human colon cancer tissue and cells, and its increased expression is positively correlated with increased pathological grading. Furthermore, increased COPB2 gene expression has been reported in six human CRC cancer cell lines [25]. In this study, we altered the expression of COPB2 in CRC cells to better characterize the roles of COPB2 in colon cancer. Knockdown of COPB2 was shown to inhibit the proliferation and induce apoptosis in RKO and HCT116 cells. Furthermore, the effects of COPB2 on cells apoptosis appeared to be associated with the activation of the JNK/c-Jun apoptotic signaling pathway. This study reveals that COPB2 is essential for the proliferation and apoptosis of colon cancer indicating that it may serve as a potential target for the treatment of CRC.

## Materials and methods

### Public database analysis

The expression of COPB2 in colorectal cancer and the clinical data was obtained by TCGA database (http://cancergenome.nih.gov/) [26]. The effect of COPB2 expression on clinicopathologic characteristics and overall survival in colorectal cancer patients was analyzed.

### Immunohistochemistry (IHC) and scoring

The expression of COPB2 in 41 colon cancer specimens and paracancerous tissues were investigated. The samples were obtained from Gansu Provincial Hospital (Gansu, China) during 2016–2017. None of the patients have received radiotherapy or chemotherapy before sample collection. Ethics committee approval was obtained from the Ethics Committee of Gansu Provincial Hospital. Immunohistochemistry was used to detect the expression of COPB2 protein as described previously [27]. The positive ratio is expressed as follows: The proportions of positive cells scores (‘0’, no staining; ‘1’, <1/3 stained; ‘2’, 1/3-2/3 stained; ‘3’, >2/3 stained) and the staining intensity scores (‘0’, none; ‘1’, weak; ‘2’, intermediate; ‘3’, strong) were added to obtain the final scores, which ranged from 0 to 6 [28].

### Cell lines and culture

COPB2 gene expression has been detected in six human colorectal cancer cells lines (RKO, SW480, HCT116, DLD1, HT-29, and SW620) in the previous research [25]. In this experiment, RKO [29–31] (Catalogue number: GCC-IN0002RT/GCC-IN0002CS) and HCT116 [32–34] (Catalogue number: GCC-IN0003RT/GCC-IN0003CS) cells were used. These cells were obtained from the Shanghai Genechem (Shanghai, China) and were cultured in RPMI 1640 medium (HyClone; GE Healthcare Life Science, Logan, UT, USA) supplemented with 10% FBS (R&D Systems, Inc., Minneapolis, MN, USA), L-glutamine, and 100 U/ml penicillin/streptomycin (Gibco; Thermo Fisher Scientific, Inc., Waltham, MA, USA) at 37°C in a 5% CO_2_ incubator.

### COPB2 knockdown

shCOPB2 (AGATTAGAGTGTTCAATTA) and shCtrl (TTCTCCGAA CGTGTCACGT) were obtained from Shanghai Gene Pharma (Shanghai GenePharma Co., Ltd., Shanghai, China). The sequences were inserted into GV248 lentiviral vectors. The shRNA vectors were transfected into CRC cells according to the manufacturer’s instructions. Briefly, RKO and HCT116 cells were seeded into 6-well plates and incubated at 37°C in an incubator with 5% CO_2_. After 24 h, lentiviruses with GV248-COPB2-shRNA or control shRNA were added per the manufacturer’s instructions. 96 h post-infection, the infected cells were observed under a fluorescence microscope (100x magnification, Olympus, Tokyo, Japan) by counting the cells with green fluorescence protein (GFP) with naked eyes. The efficiency of COPB2 knockdown was characterized via Western blotting and the experiment was repeated 3 times.

### CCK8 cell proliferation assay

CCK8 (Dojindo, Kumamoto, Japan) assays were used to characterize the proliferation of RKO and HCT116 cells. Parental cells (uninfected RKO or HCT116 cells), shCOPB2-treated cells, and shCtrl-treated cells were seeded into 96-well plates. After incubation for 24, 48, 72, 96, and 120 h, each well was supplemented with CCK8 reagent per the manufacturer’s instructions, and the optical density (OD) at a wavelength of 450 nm was measured. The experiment was repeated 3 times.

### Celigo cell count

Parental cells, shCOPB2-treated cells, and shCtrl-treated cells were seeded into 96-well plates. After 24h, a Celigo Image Cytometer (Nexcelom, Lawrence, MA USA) was used to measure the number of green fluorescent cells in each plate once a day for 5 days. Cell proliferation curves were subsequently generated.

### Apoptosis analysis

Flow cytometry was used to determine the cellular apoptosis rate. Parental cells, shCOPB2-treated cells and shCtrl-treated cells were seeded onto 6-well plates and grown to 70% confluence. The cells were harvested, washed with PBS, and then centrifuged at 1300 rpm for 5 min. 1x binding buffer was used to wash the cells, which were then centrifuged at 1300 rpm for 3 min. Cell pellets were then resuspended in 200 μl 1x binding buffer. Ten microliters of Annexin V-APC was added, and the cells were incubated for 10–15 min for staining. A flow cytometer (Guava EasyCyte HT, USA) was used to determine the apoptosis rate. The experiment was repeated 3 times.

### Caspase 3/7 activity analysis

Parental cells, shCOPB2-treated cells and shCtrl-treated cells were seeded in 96-well plates and incubated at 37°C in a 5% CO_2_ incubator for 72 h. Caspase-Glo (Caspase-Glo^®^ 3/7 Assay, G8091, Promega, Madison, WI, USA) reaction solution was prepared according to the manufacturer’s instructions. Cells were collected, washed with PBS and seeded in 96-well plates. A total of 100 μl Caspase-Glo reaction solution was added per well. The plates were then incubated at room temperature for 1–2 h, and the fluorescence intensity was subsequently measured. The experiment was repeated 3 times.

### Western blot analysis

Western blot analysis was performed to measure the levels of apoptosis-related proteins in parental cells, shCOPB2-treated cells, and shCtrl-treated cells as previously described. Brieflf, total proteins from RKO and HCT116 cells were extracted, separated using 10% SDS-PAGE, and then transferred to polyvinylidene difluoride (PVDF) membranes (EMD Millipore, Bedford, UK). After blocking in 5% skim milk at 37°C for 1 h, the membranes were incubated with various antibodies, including antibodies againse COPB2 (Sigma-Aldrich, HPA036867), BCL-2 (Abcam, ab185002), Bax (Abcam, ab182734), c-Jun (Abcam, ab32137), JNK (Abcam, ab179461), p53 (CST, #2527), Bad (Abcam, ab32445), Caspase3 (Abcam, ab32042) and GAPDH (Abcam, ab181602) overnight at 4°C. Then the membranes were subsequently washed 3 times with TBST and incubated with fluorescently labeled secondary antibodies for 1–2 h at room temperature. Bands were visualized using an Odyssey detection system (Licor Biosciences, Nebraska, USA). The experiment was repeated 3 times.

### Statistical analysis

GraphPad Prism v5.0 and SPSS 16.0 software systems were used for all statistical analyses. Data are presented as the means±SD. Comparisons between two groups of samples were performed via Student’s t-test, and comparisins among three groups were performed via one-way ANOVA followed by Turkey’s or Dunn’s posthoctest. Kaplan-Meier curve was applied to survival analysis. Values *P*<0.001, *P*<0.01, *P*<0.05 were defined as statistically significant.

## Results

### The relationship between COPB2 expression and clinical characteristics in colorectal cancer

**Fig 1:**
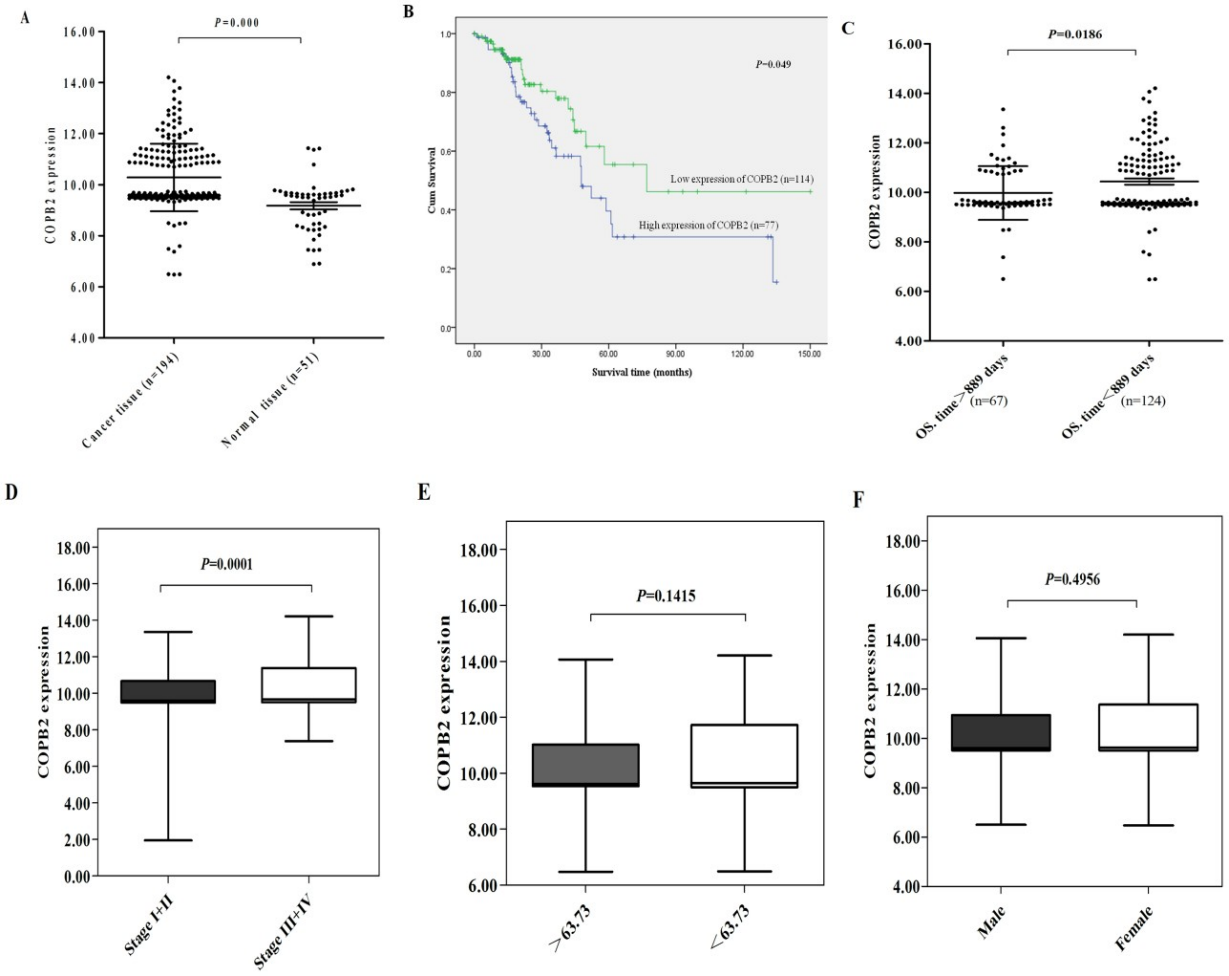
The correlation between COPB2 expression and clinical data of colorectal cancer patients. (A) The expression of COPB2 in colorectal cancer tissues was higher than that in paracancerous tissues. ***P<0.001. (B) Kaplan-Meier curves demonstrated that the OS of patients with higher expression of COPB2 is lower than that of patients with lower expression. *P<0.05. (C) The expression of COPB2 in patients with OS. time > 889 days was lower than that in patients with OS. time < 889 days. (D) Increased expression of COPB2 was correlated significantly with pathological stage of colorectal cancer. ***P<0.001. (E-F) The expression of COPB2 was incorrelated with the age and gender of colorectal cancer patients (*P*>0.05).

The clinical data of 194 colorectal cancer tissues and 51 paracancerous tissues were obtained from TCGA database. Compared to paracancerous tissues, the expression of COPB2 was up-regulated in tumor tissues (***P<0.001, Fig 1A). The result suggested that COPB2 may be a oncogene in colorectal cancer. The relationship between COPB2 expression and the overall survival was analyzed by using the clinical data of 194 cancer patients. The patients with missing OS data were aliminated. The Kaplan-Meier analysis was applied for the overal survival of remaining patients. The results showed that the OS of patients with high expression of COPB2 was lower than that of patients with its lower expression of COPB2 (*P<0.05, Fig 1B). The average OS. time of 191 patients was 889 days. It was found that the expression of COPB2 in patients with OS. time > 889 days was lower than that in patients with OS. time < 889 days. (*P<0.05, Fig 1C). To investigate the correlation between COPB2 expression and clinical variables of colorectal cancer patients, the data from TCGA were analyzed. As shown in Fig 1D–1F, the expression of COPB2 was correlated with the pathologic stage of colorectal cancer patients, but incorrelated to age and gender.

### COPB2 is highly expressed in colorectal cancer tissues and positively correlated with pathological stages of patients

**Fig 2:**
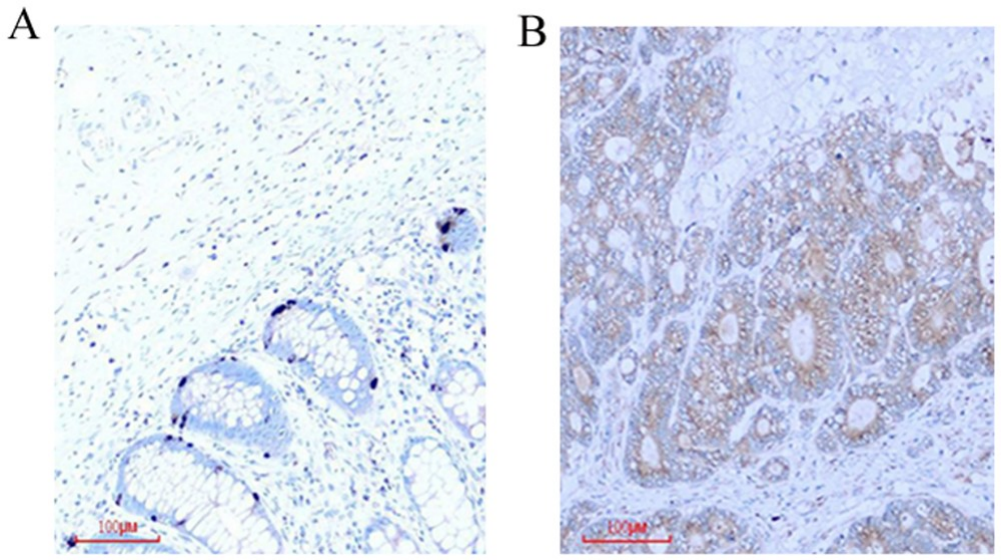
Immunohistochemical analysis of COPB2 in colorectal cancer specimens. (A) COPB2 was decreased expression in adjacent tissues. (B) Higher expression of COPB2 in cancer tissues.

To further confirm the correlation between COPB2 and colorectal cancer, IHC was used to investigated COPB2 expression in 41 cases of colorectal cancer tissues and paracancerous tissues. The results demonstrated that the staining of COPB2 in cancer tissues was markedly stronger than that in adjacent noncancerous tissue (Fig 2A and 2B). The final scores of COPB2 protein density were 4.8293±1.0932 in cancer tissues and 2.1220±1.0999 in paracancerous tissues (Table 1, ***P<0.001). We analyzed the clinicopathological parameters of 41 patients with colorectal cancer, including age, gerder, tumor diameter and pathological stages. The results indicated that the expression of COPB2 was significantly correlated with the pathological stages and tumor differentiation. The expression of COPB2 in stages III and IX was higher than that in stages I-II. The lower degree of tumor differentiation was the stronger COPB2 expressed (Table 2, *P<0.05 or ***P<0.001). These results were consistent with the data from TCGA database.

**Table 1:** Expression of COPB2 in colorectal cancer and paracancerous tissue by IHC staining.

Tissue	*n*	Mean±SD	*P*-value
Cancer tissue	41	4.8293±1.0932	*P* = 0.000
Paracancerous tissue	41	2.1220±1.0999	

**Table 2:** Relationship between COPB2 expression and clinicopathological parameters by IHC staining.

Clinicopathological parameters	*n*	Mean±SD	*P*-value
**Ages(years)**			
≥60	22	5.0362±1.2019	*P* = 0.4010
<60	19	4.6842±1.0569	
**Gender**			
Male	21	5.0012±0.7947	*P* = 0.2970
Female	20	4.6500±1.3485	
**Tumor diameter**			
≥5	23	4.6967±0.3333	*P* = 0.2610
<5	18	5.0656±0.1891	
**Tumor differentiation**			
high	9	3.8229±1.36423	***P = 0.000
medium	11	4.4545±0.68755	
low	21	5.4286±0.7464	
**Pathological grading**			
I+II	18	4.3370±1.0209	*P = 0.02
III+IV	23	5.5634±0.7048	

### COPB2 knockdown in RKO and HCT116 cells via lentivirus—Mediated RNAi silencing

**Fig 3:**
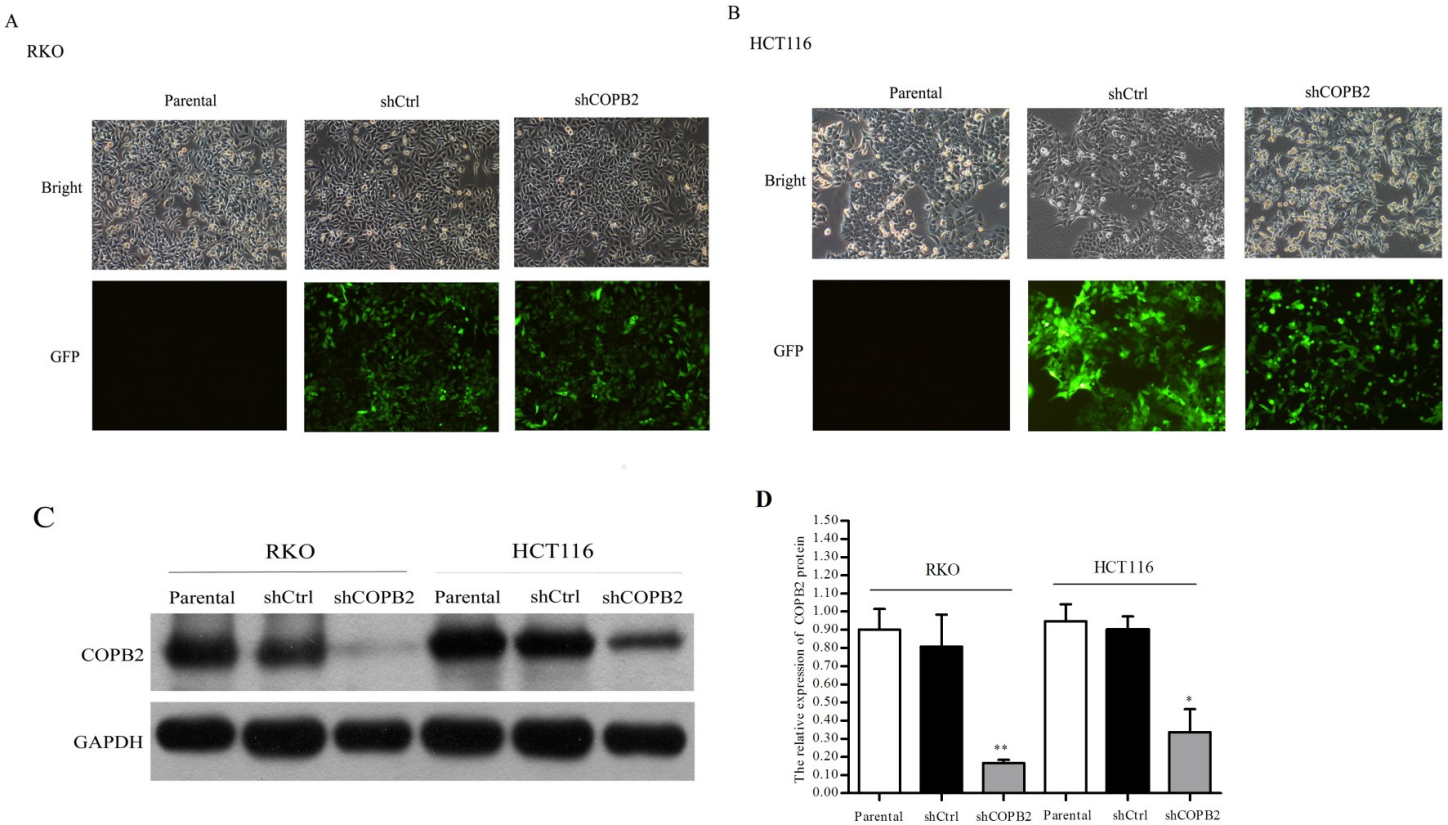
Lentivirus -mediated siRNA treatment decreased COPB2 expression in RKO and HCT116 cells. (**A-B**) The transduction efficiency was assessed via light and fluorescence microscopey after infection at MOIs of 5 and 15. Upper images: light microscopy; lower images: fluorescence microscopy; (100x magnification). (**C-D**) COPB2 protein expression was markedly inhibited by lentivirus—mediated RNAi silencing. **P<0.01, *P<0.05.

COPB2 expression in RKO and HCT116 cells was targeted using lentiviral vectors. Infection rates were assessed by fluorescence imaging (Fig 3A and 3B). Based on GFP fluorescence, more than 80% of cells were infected. Western blot assays showed that the protein levels of COPB2 were significantly reduced in shCOPB2-treated cells compared with those in shCtrl-treated cellss and parental cells (**P<0.01 or *P<0.05, Fig 3C and 3D). Thus, the expression of COPB2 in RKO and HCT116 cells was markedly reduced via lentivirus—mediated RNAi silencing.

### COPB2 knockdown inhibits the proliferation of RKO and HCT116 cells

**Fig 4:**
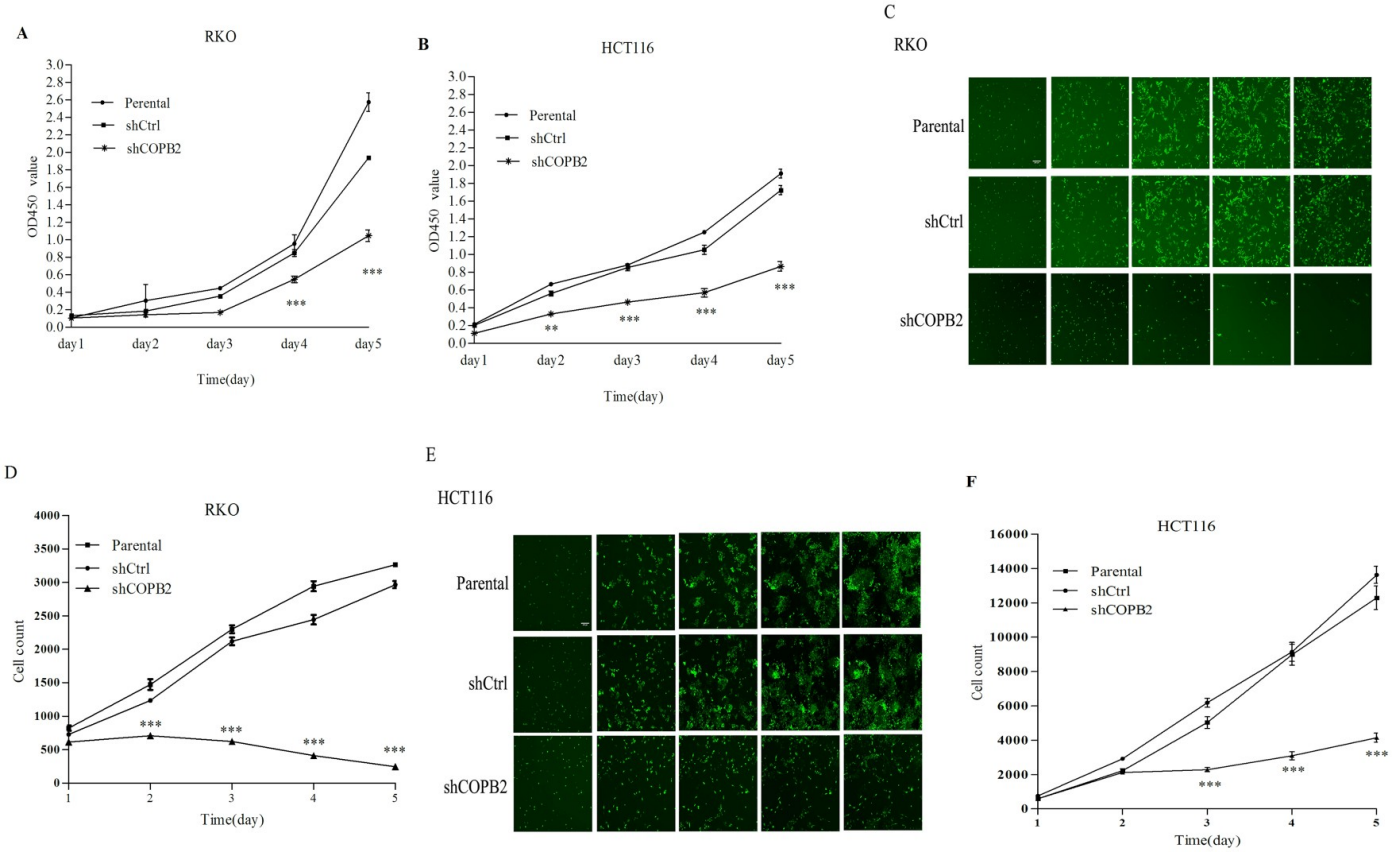
COPB2 knockdown suppresses the proliferation of RKO and HCT116 cells. (**A-B**) The proliferative capacity of parental cells, shCtrl-treated cells, and shCOPB2-treated cells were measured via CCK8 assays. After incubation for 48 or 96 hours, the proliferative capacity of shCOPB2-treated cells were decreased in RKO and HCT116 cells. (**C-F**) Celigo cell counting assays indicated that the proliferation was significantly reduced when COPB2 expression was inhibited. Data are shown as the mean ± SD. **P<0.01, ***P<0.001, compared with shCtrl and parental cells.

To characterize the functional role of COPB2 in RKO and HCT116 cell proliferation, CCK8 assays and Celigo cell counting assays were performed. The CCK8 assays demonstrated that, compared with that in the parental cells and shCtrl-treated cells, the proliferation ability was decreased in shCOPB2-treated cells after culturing for 2 or 4 days (**P<0.01 or ***P<0.001, Fig 4A and 4B). Celigo cell counting assays also demonstrated that the proliferation of cells treated with shCOPB2 was significantly inhibited 2 or 3 days after infection compared with that of control cells (Fig 4C–4F). These results indicate that COPB2 knockdown inhibited the proliferation of RKO and HCT116 cells.

### COPB2 knockdown promotes the apoptosis of RKO and HCT116 cells

**Fig 5:**
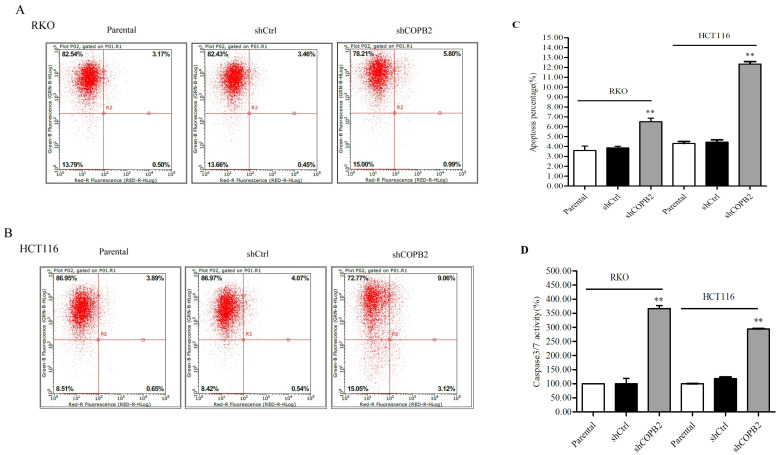
COPB2 knockdown promotes apoptosis of RKO and HCT116 cells and increases Caspase 3/7 activity. (**A-C**) Flow cytometry analysis indicated that COPB2 knockdown significantly promoted cells apoptosis in RKO and HCT116 cells. (**D**) Caspase 3/7 activity was significantly increased in shCOPB2-treated groups of RKO and HCT116 cells. Data are represented as the means ± SD. **P<0.01 compared with the shCtrl and parental cells groups.

To characterize the effects of COPB2 knockdown on RKO and HCT116 cells apoptosis, flow cytometry and Caspase 3/7 activity analysis were performed with parental cells, shCtrl-treated cells and shCOPB2-treated cells. Flow cytometry analysis revealed that the apoptosis rates of the parental groups, shCtrl-treated groups, and shCOPB2-treated groups of RKO cells were 3.59%, 3.87% and 6.52%, respectively. The apoptosis rates of in HCT116 cells were 4.31%, 4.44% and 12.34%, respectively. It suggests that the apoptosis rates were increased for shCOPB2-treated cells compared with those for parental cells and shCtrl-treated cells (**P<0.01, Fig 5A–5C). Caspase 3/7 activity analysis additionally indicated that the Caspase 3/7 activity was increased significantly after COPB2 knockdown (**P<0.01, Fig 5D). Taken together, these results indicate that COPB2 knockdown promoted apoptosis in RKO and HCT116 cells.

### COPB2 knockdown alters the expression of apoptosis-related proteins in RKO and HCT116 cells

**Fig 6:**
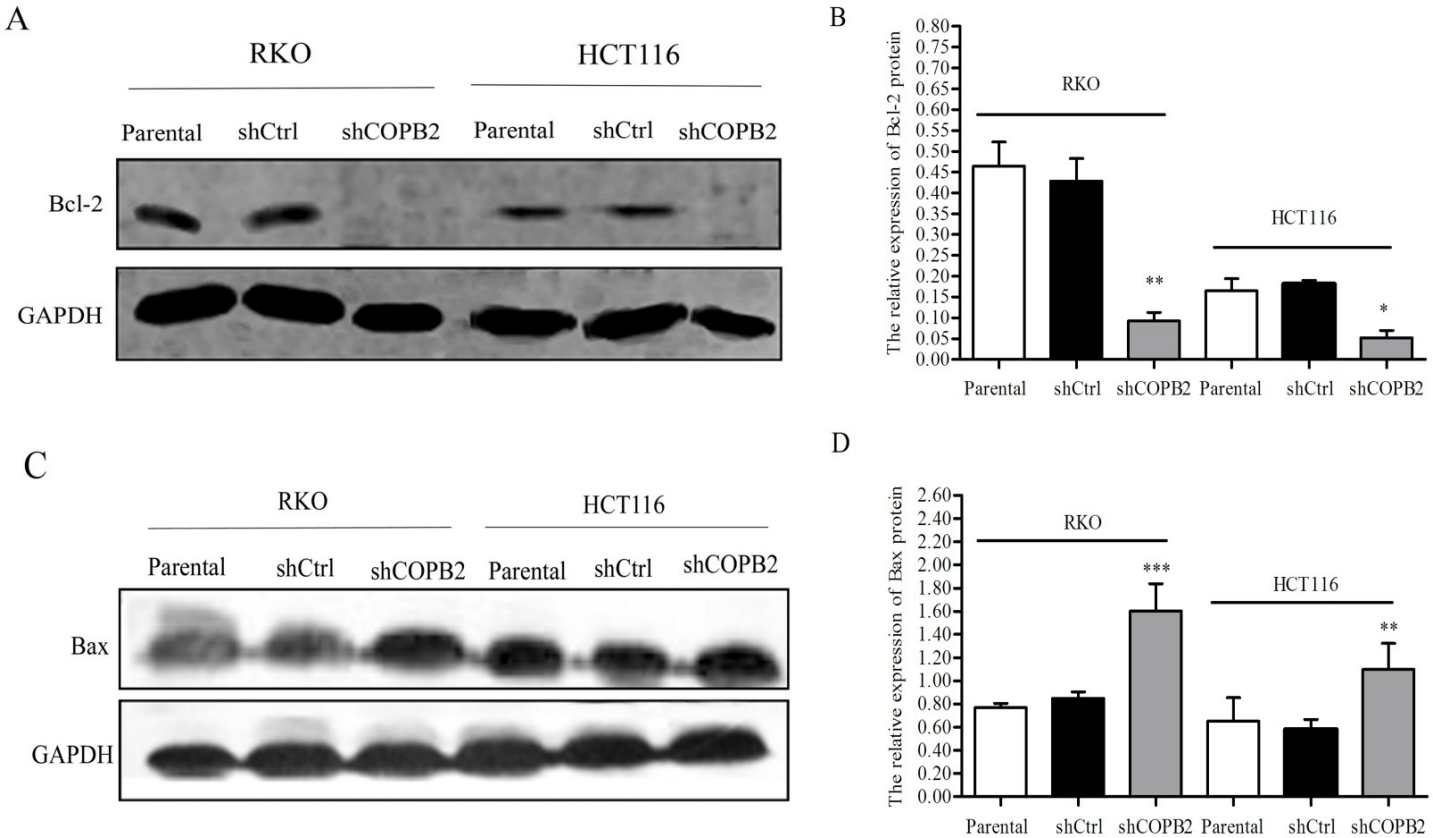
Western blot analysis of apoptosis-related proteins expression in RKO and HCT116 cells. (**A-B**) Following knockdown of COPB2, the protein expression level of Bcl-2 was reduced in RKO and HCT116 cells, ***P<0.001 or *P<0.05 compared with shCtrl and parental cells groups. (**C-D**) In contrast, the expression level of Bax was increased in RKO and HCT116 cells. ***P<0.001 or **P<0.01 compared with shCtrl and parental cells groups.

Western blot analysis indicated that the expression of Bcl-2 was decreased significantly after COPB2 knockdown (***P<0.001 or *P<0.05, Fig 6A and 6B). In contrast, the expression of Bax was found to be increased in RKO and HCT116 cells after COPB2 knockdown (***P<<0.001 or **P<0.01, Fig 6C and 6D).

### COPB2 knockdown induces apoptosis of RKO and HCT116 cells via the JNK/c-Jun signaling pathway

**Fig 7:**
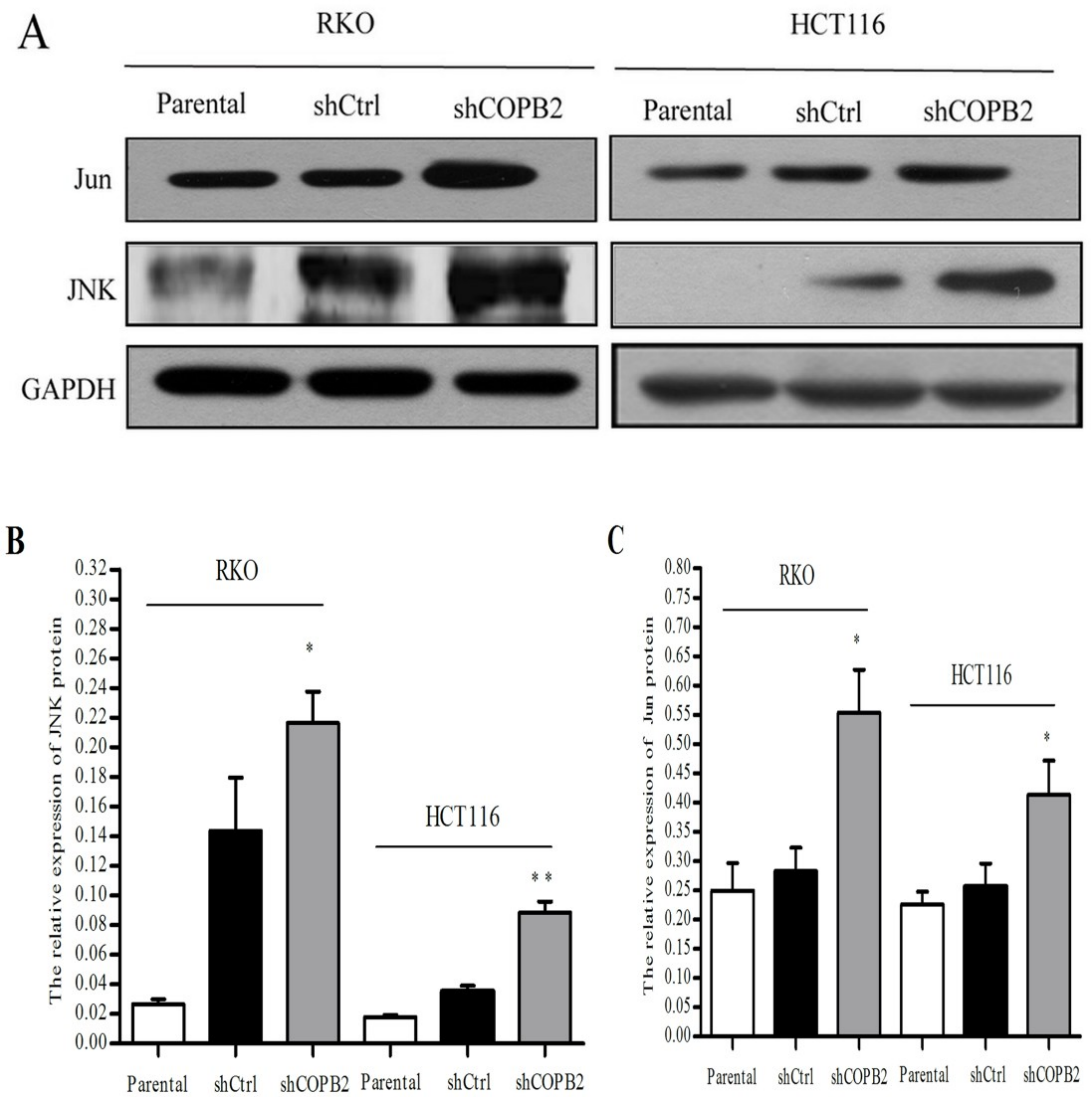
Western blot analysis of JNK and Jun protein expression in RKO and HCT116 cells. (**A-C**) COPB2 knockdown increased the expression levels of JNK and Jun proteins, *P<0.05 or **P<0.01 compared with shCtrl and parental cells.

**Fig 8:**
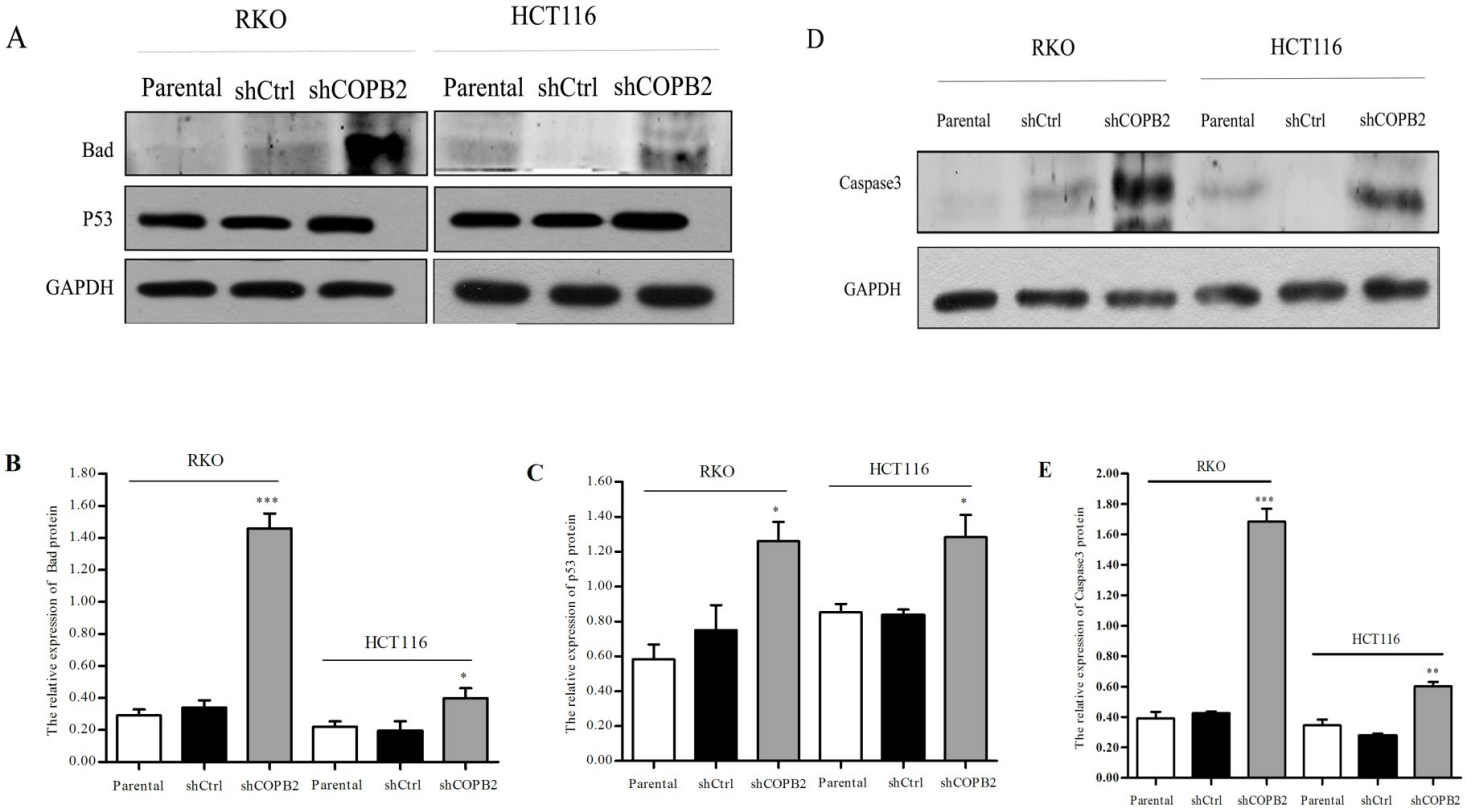
Western blot analysis of the expression levels of JNK/c-Jun signaling pathway related proteins in RKO and HCT116 cells. (**A-E**) COPB2 knockdown increased the protein expression levels of Bad, p53, and Caspase 3. *P<0.05, **P<0.01, or ***P<0.001 compared with shCtrl and parental cells.

To further characterize the potential correlation between apoptosis induced by COPB2 knockdown and the JNK/c-Jun signaling pathway in RKO and HCT116 cells, Western blot analysis was used to determine the expression of proteins related to the JNK/c-Jun signaling pathway. As shown in Fig 7A–7C, the protein expression levels of JNK and Jun were increased after silencing of COPB2, suggesting that COPB2 gene silencing may be accompanied by activation of the JNK/c-Jun apoptosis signaling pathway in RKO and HCT116 cells. Following activation of JNK/c-Jun signal pathway, there are generally two ways to induce the apoptosis of tumor cells: increasing the expression of pro-apoptotic proteins, such as p53 and Bad, and inducing alterations within mitochondria, including activation of the binding of related proteins like Bax and cytochrome C to subsequently act on Caspase3 to cause apoptosis by binding with apoptosis-related substrates. Therefore, we further determined the expression levels of proteins related to this pathway, such as Bad, p53, and cleaved Caspase 3. As demonstrated by Western blot analysis (Fig 8A–8E), we determined that compared with that in parental cells and shCtrl-treated cells, the expression levels of Bad, p53 and cleaved Caspase 3 proteins were increased in shCOPB2-treated cells.

## Discussion

As CRC research continues to advance, cancer biotherapy has become an increasingly promising treatment in addition to surgery, chemotherapy and radiotherapy. Among biotherapies molecularly targeted therapy and gene therapy have achieved positive results [35,36]. In light of these promising results, identifying new gene targets for CRC biotherapy is of increasing importance. Vesicular transport is a critical means of intracellular substance transport. Coat proteins (COPs) play an important role in vesicle transport [37] and include three types of proteins: Clathrin, COPBI and COPBII. COPBI contains seven subunits: α-COP, β-COP, β'-COP, γ-COP, δ-COP, ε-COP, and ζ-COP [38–41]. Coatomer protein complex subunit, beta 2 (COPB2) is one of these seven subunits and participates in the process of membrane transport between the endoplasmic reticulum and Golgi apparatus [19,22,42–45]. Mi Y et al. have reported that COPB2 is highly expressed in human prostate cancer and promoted the proliferation of prostate cancer cells [24]. Many studies have found that COPB2 was up-regulated in various human tumors, such as breast cancer, gastric cancer [46] and glioma [47].

Is COPB2 also related to the occurrence and development of colorectal cancer? TCGA database was used to confirm the correlation between COPB2 and clinical parameters of colorectal cancer. In addition, we collected 41 clinical specimens, and detected the expression of COPB2 in cancer tissues and adjacent tissues by immunohistochemistry. The results confirmed that COPB2 was closely related to the differentiation degree, tumor stages and a shorter survival time of colorectal cancer. Then, we seriously suspected that COPB2 may be an oncogene in the development of colorectal cancer.

To further investigate the role of COPB2 gene in the development of colorectal cancer, we suppressed the expression of COPB2 via lentivirus-medicated RNAi transduction in CRC RKO and HCT116 cells. CCK8 assays and Celigo cell counting assays confirmed that compared with control groups, the silence of COPB2 significantly decreased the proliferation ability of RKO and HCT116 cells. Annexin V is a Ca^2+^ dependent phospholipid protein, which can be combined with phosphoestersene. Labeling Annexin V with fluorescein can be used to detect the early apoptosis of cells [48]. Caspase is a group of cysteine proteases located in cytosol, which acts as an initiator and executor in the process of apoptosis [49]. Herein, the activity of caspase3/7 was detected to reflect the apoptosis of the cells. The results showed that the proportion of natural apoptosis and the activity of caspase-3/7 increased after the silencing of COPB2 gene compared with the control cells, It suggested that COPB2 gene silencing could inhibit CRC cell proliferation and induce apoptosis.

Apoptosis is regulated by a variety of apoptosis-related proteins, which can be divided into two categories: pro-apoptotic proteins and anti-apoptotic proteins. Bcl-2 family is a family of apoptosis related proteins, including Bcl-w, Bcl-x, Bax, Bak, bad, Bim and so on. As an anti-apoptotic protein, Bcl-2 has an obvious inhibitory effect on the release of cytochrome C, which further inhibits the subsequent activation of Caspase protein and ultimately inhibits cell apoptosis [50]. Different from Bcl-2 protein, Bax protein is a pro-apoptotic protein. When the expression of Bax protein was increased, apoptosis will increase [51,52]. In this study, after the silence of COPB2 gene, the expression of Bcl-2 was significantly decreased, while the expression of Bax was enhanced. It suggested that COPB2 gene silencing may induce tumor cell apoptosis through the mitochondrial pathway.

Activation of the JNK/c-Jun signaling pathway is one of the most critical ways to induce cellular apoptosis. The activation of this pathway can increase the expression of pro-apoptotic proteins, such as p53 and Bad. At the same time, it can also activate mitochondria by promoting the binding of Bax and Cytochrome c and ultimately acting on Caspase3 to cause apoptosis by binding with apoptosis-related substrates. In our study, Western blot analysis further demonstrated that the expression levels of JNK and Jun proteins were increased after the silencing of the COPB2 gene. It suggestd that COPB2 gene silencing may activate the JNK/c-Jun apoptotic signaling pathway to induce apoptosis in CRC cells. We further analyzed the expression levels of various JNK/c-Jun pathway-related proteins, such as p53, Bad, and cleaved Caspase3 via Western blot analysis. Compared with the control groups, we found that the expressions of p53 and Bad increased when COPB2 was silenced. Caspase-3 plays an irreplaceable role in apoptosis, and it can be blocked by Bcl-2. In the process of apoptosis, pre-caspase 3 is activated and then cracked into cleaved caspase 3 to promote apoptosis. In current research, we detected cleaved caspase 3 by WB and found that the expression of cleaved caspase 3 in shCOPB2 group was significantly higher than that in shCtrl or parental cells groups. It indicated that the silence of COPB2 could activate caspase 3 and transform it into cleaved caspase 3 to promote apoptosis. Our results further demonstrated that the activation of the JNK/c-Jun signaling pathway and upregulation of apoptotic protein expression were the important mechanisms of apoptosis induced by COPB2 gene silencing in RKO and HCT116 cells.

The relationship between COPB2 gene and colorectal cancer is an interesting topic. Our study has focused on the induction of CRC cell apoptosis through the JNK/c-Jun signaling pathway following COPB2 gene silencing. It is possible that there are additional signaling pathways involved, which warrants further investigation. In current study, only some basic work have been done. Next, more tissue samples of patients with colorectal cancer will be collected and analyzed. Whether there are some other signaling pathways involved in apoptosis mediated by COPB2 gene silencing will be confirmed. More important is that the function of COPB2 gene will be further verified in tumor-bearing mice.

## Conclusions

In conclusion, our study has demonstrated that COPB2 is essential for the development of CRC RKO and HCT116 cells. COPB2 gene silencing inhibits CRC cell proliferation and induces apoptosis via the JNK/c-Jun signaling pathway.

## Supporting information

S1 Fig (TIF)

S2 Fig (TIF)

S3 Fig (TIF)

S4 Fig (TIF)

S5 Fig (TIF)

S6 Fig (TIF)

S7 Fig (TIF)

S8 Fig (TIF)

S1 File (RAR)

S2 File (RAR)

S1 Raw images (PDF)
