# Supplementary figures and images for: COPB2 gene silencing inhibits colorectal cancer cell proliferation and induces apoptosis via the JNK/c-Jun signaling pathway
Source: PLoS One. 2020 Nov 19;15(11):e0240106. doi: 10.1371/journal.pone.0240106 (PMC7676692; doi:10.1371/journal.pone.0240106)

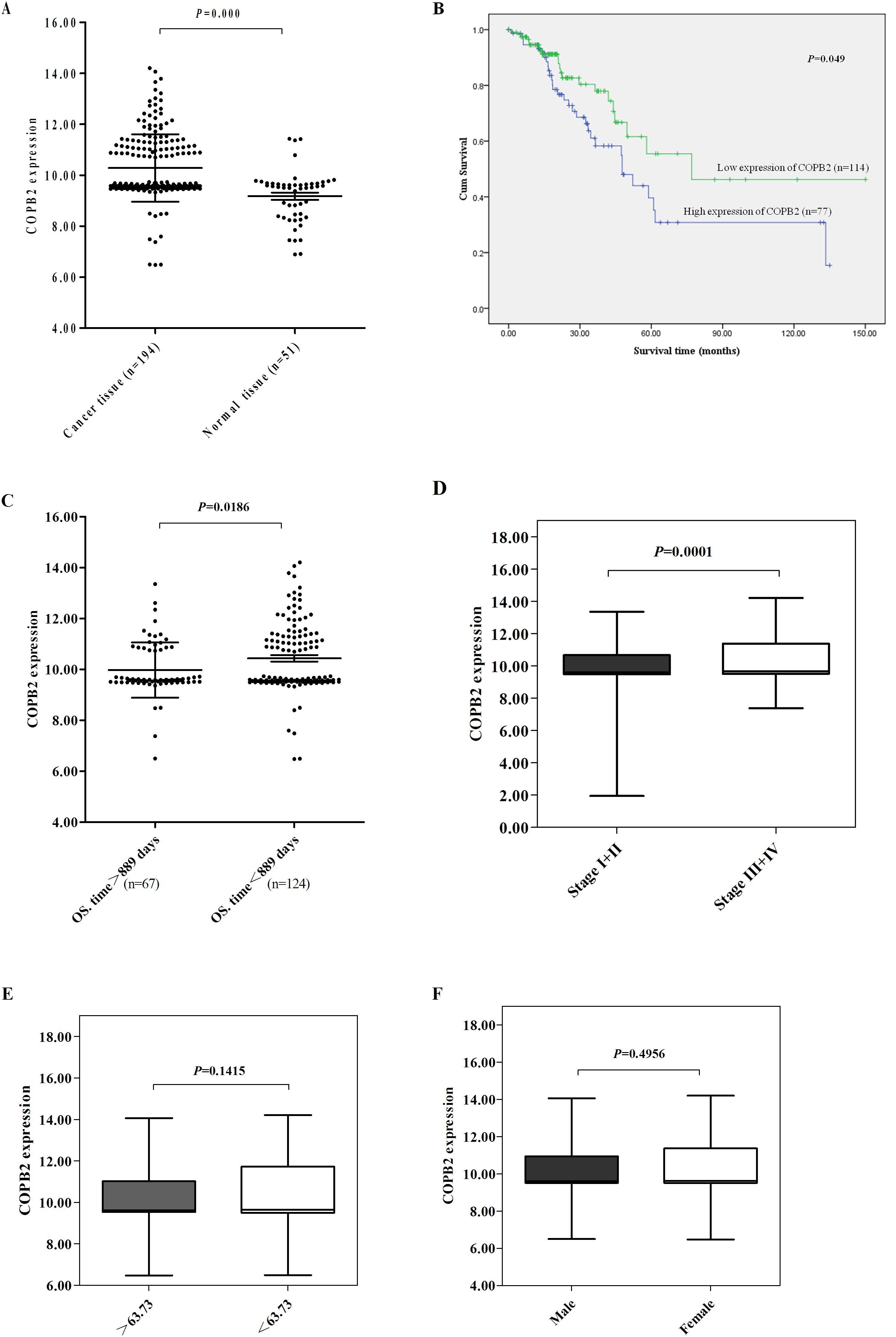

Supplement: S1 Fig — (TIF) [file pone.0240106.s001.tif]

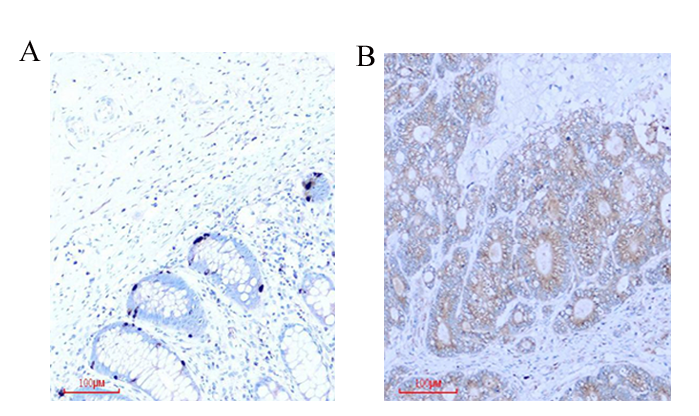

Supplement: S2 Fig — (TIF) [file pone.0240106.s002.tif]

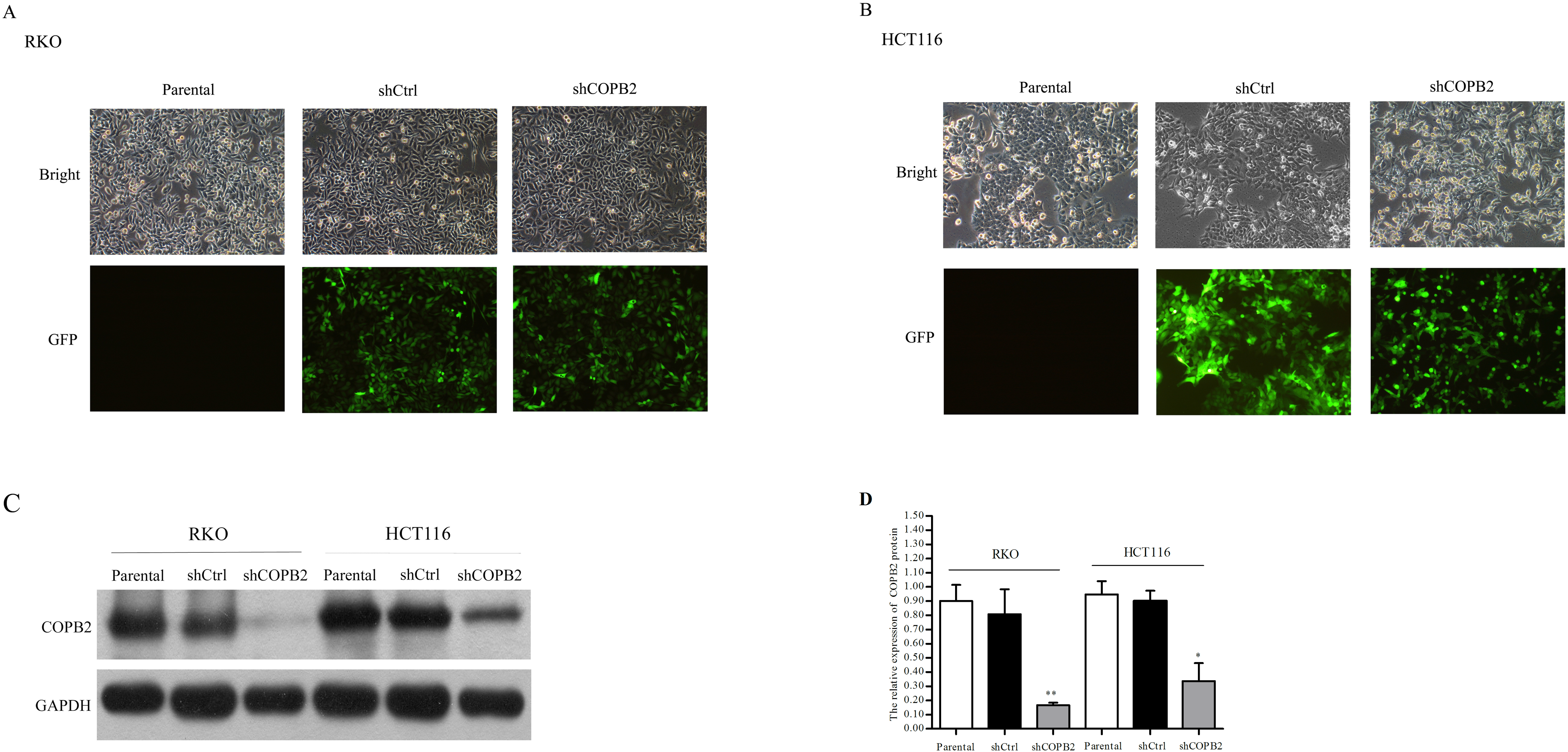

Supplement: S3 Fig — (TIF) [file pone.0240106.s003.tif]

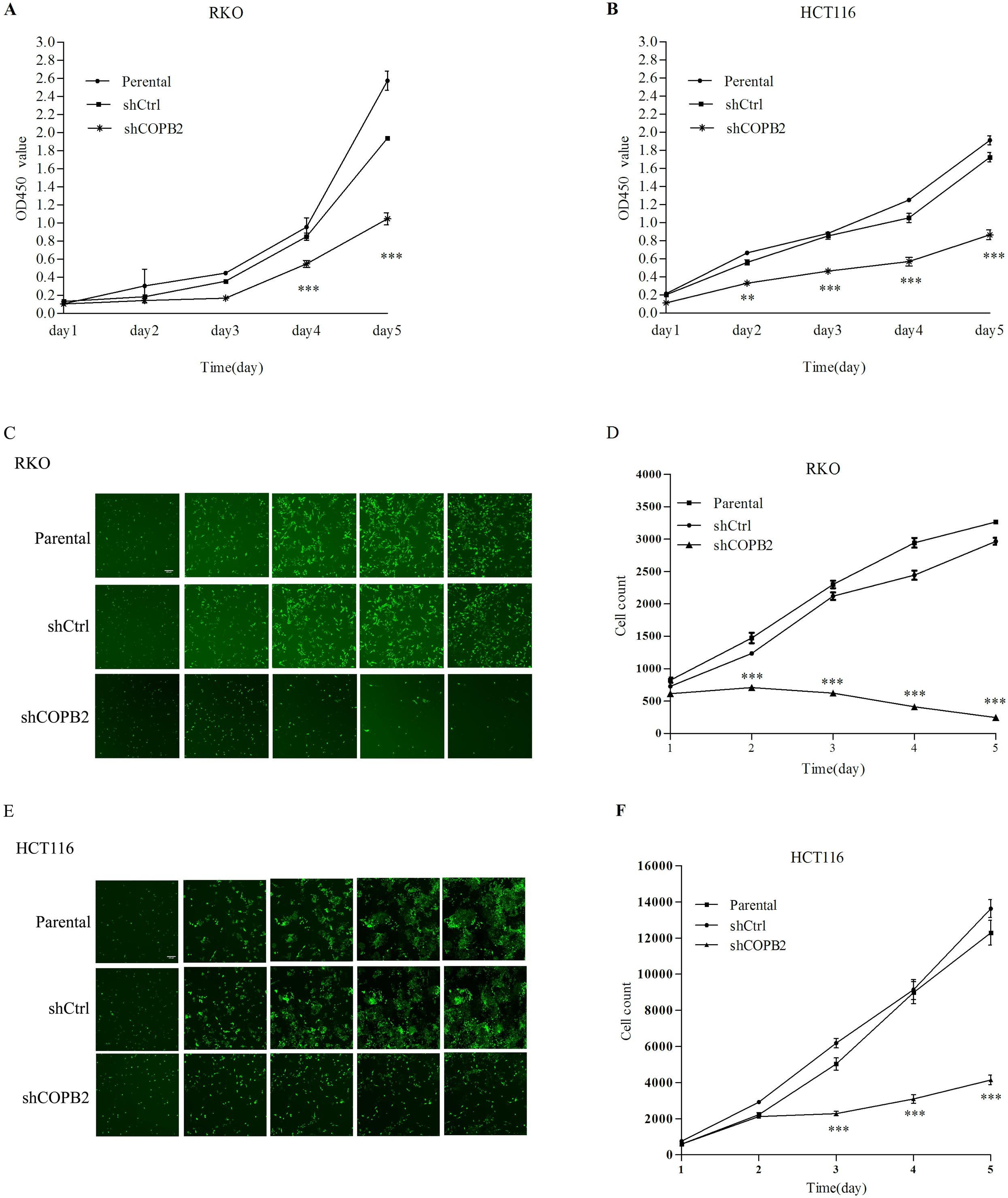

Supplement: S4 Fig — (TIF) [file pone.0240106.s004.tif]

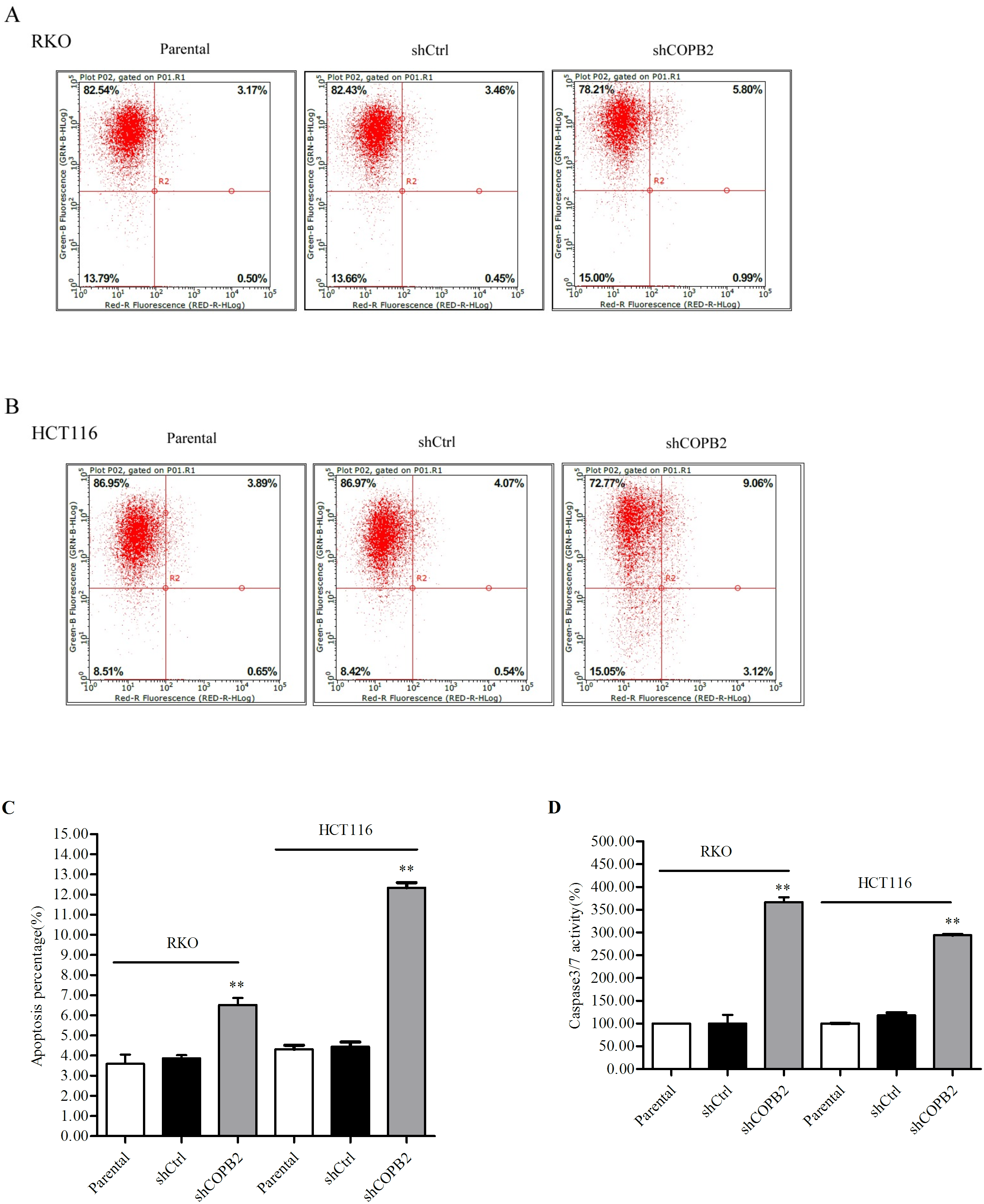

Supplement: S5 Fig — (TIF) [file pone.0240106.s005.tif]

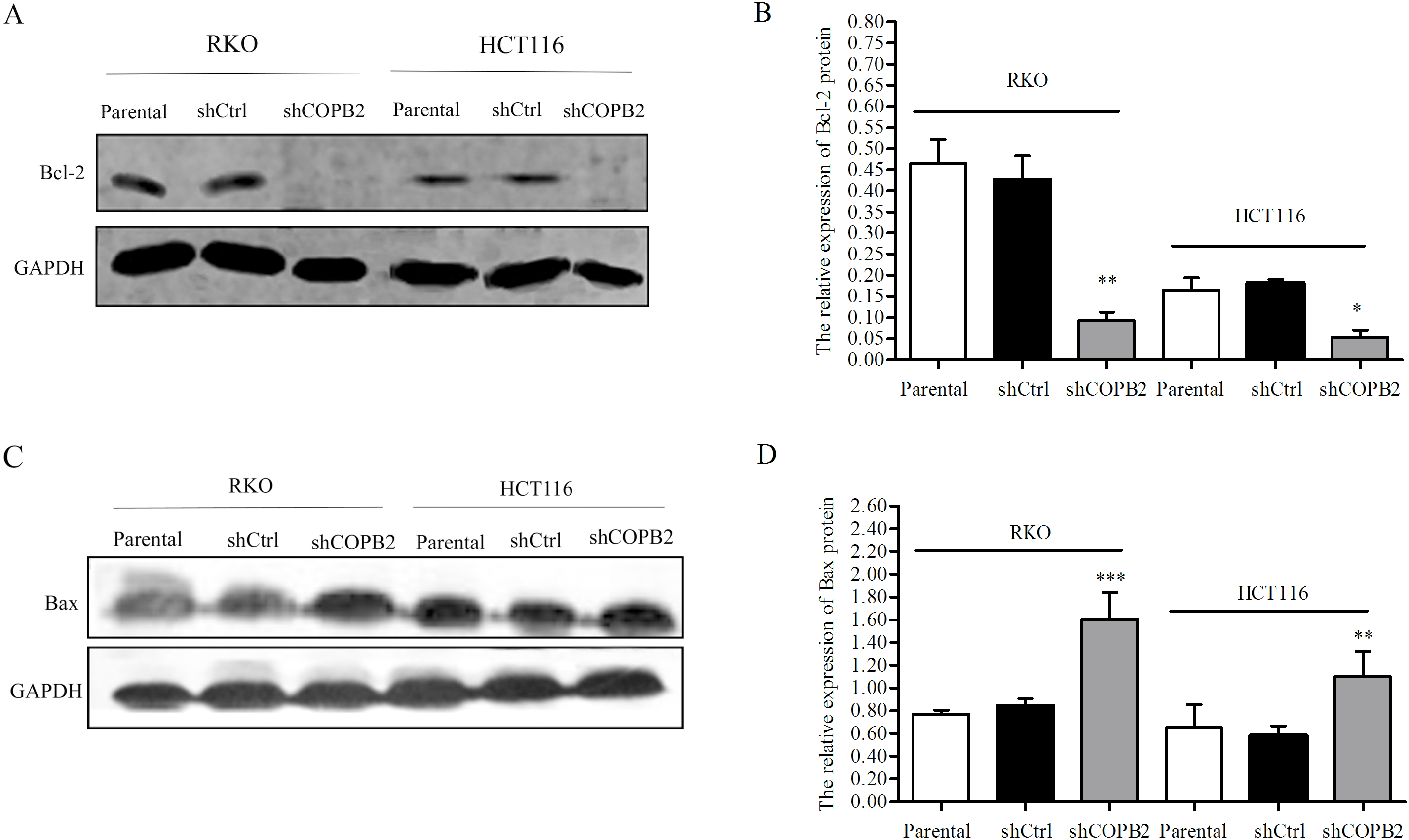

Supplement: S6 Fig — (TIF) [file pone.0240106.s006.tif]

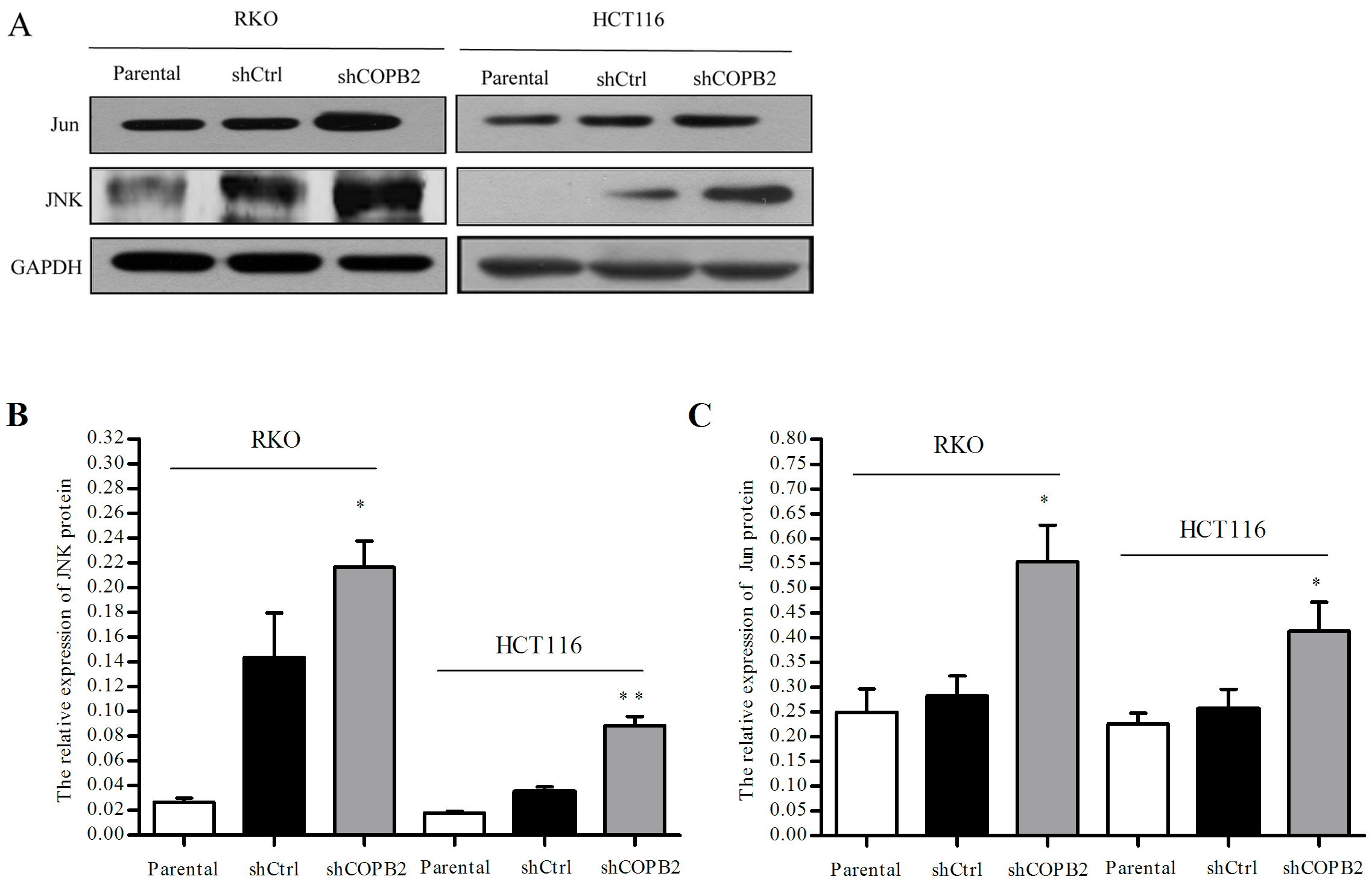

Supplement: S7 Fig — (TIF) [file pone.0240106.s007.tif]

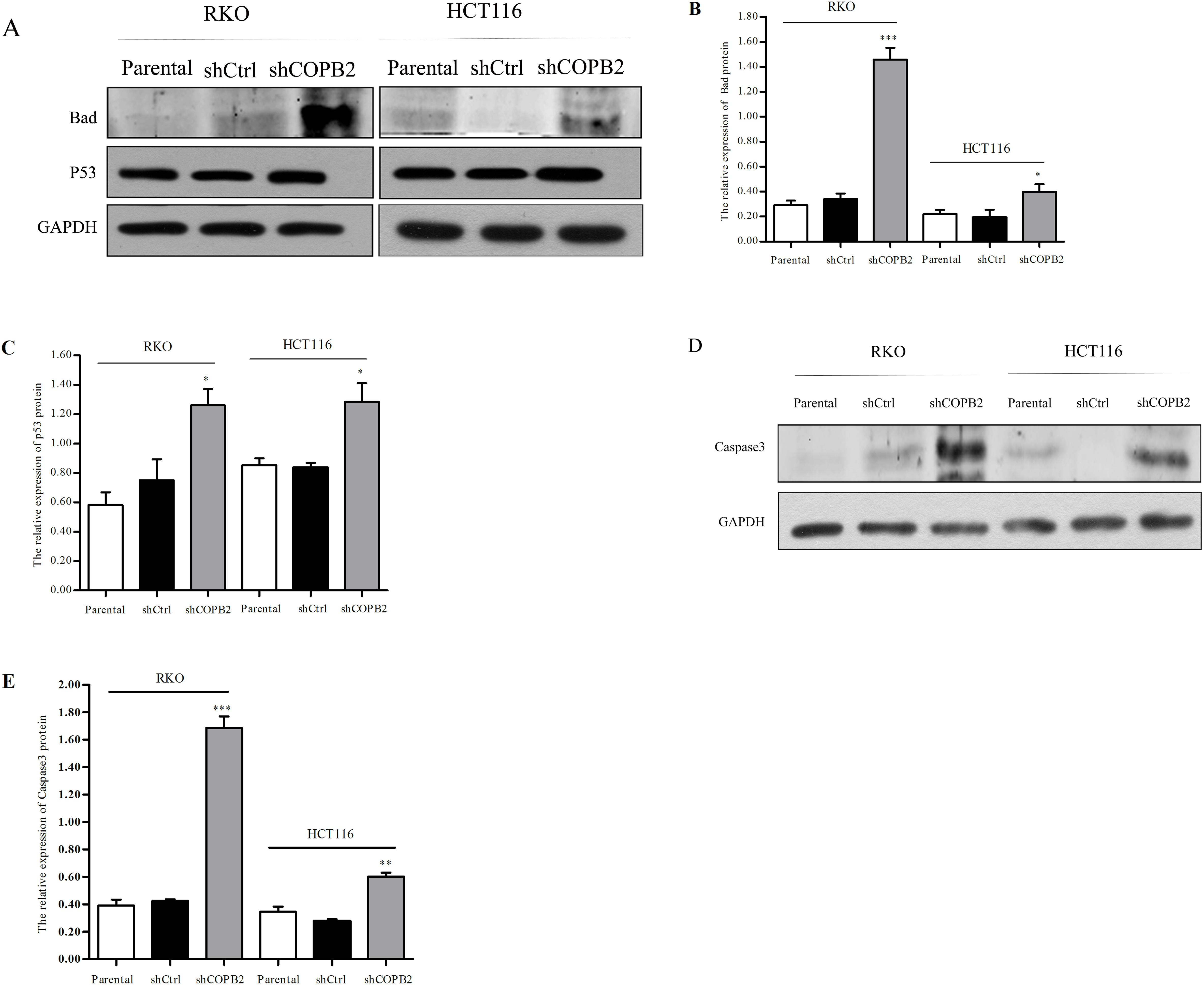

Supplement: S8 Fig — (TIF) [file pone.0240106.s008.tif]
