# Supplementary material for: COPB2 gene silencing inhibits colorectal cancer cell proliferation and induces apoptosis via the JNK/c-Jun signaling pathway
Source: PLoS One. 2020 Nov 19;15(11):e0240106. doi: 10.1371/journal.pone.0240106 (PMC7676692; doi:10.1371/journal.pone.0240106)
Supplement: S1 Raw images — (PDF) [file pone.0240106.s011.pdf]

1.Lentivirus -mediated siRNA treatment decreased COPB2 expression in RKO and HCT116 cells.

HCT116:

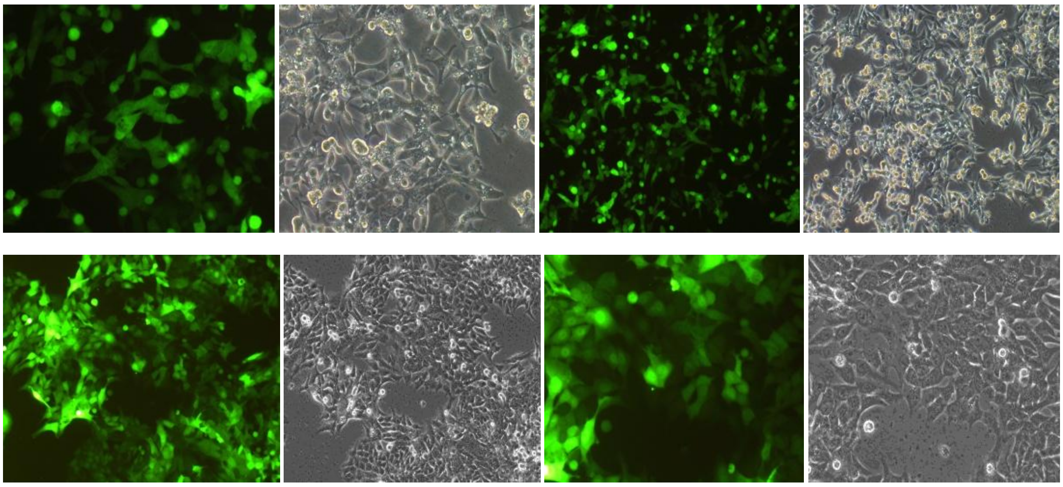

RKO:

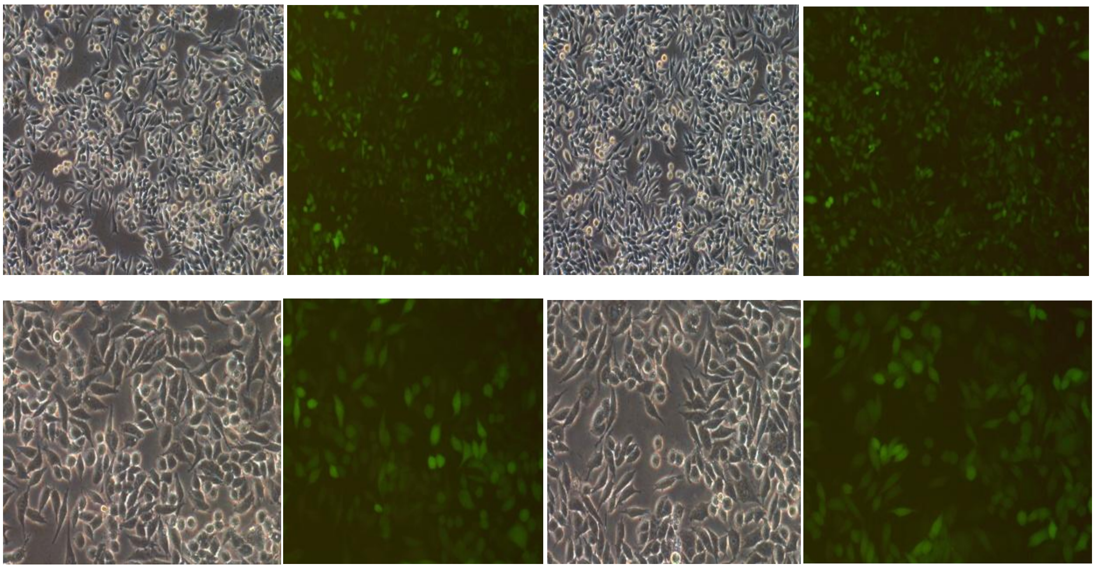

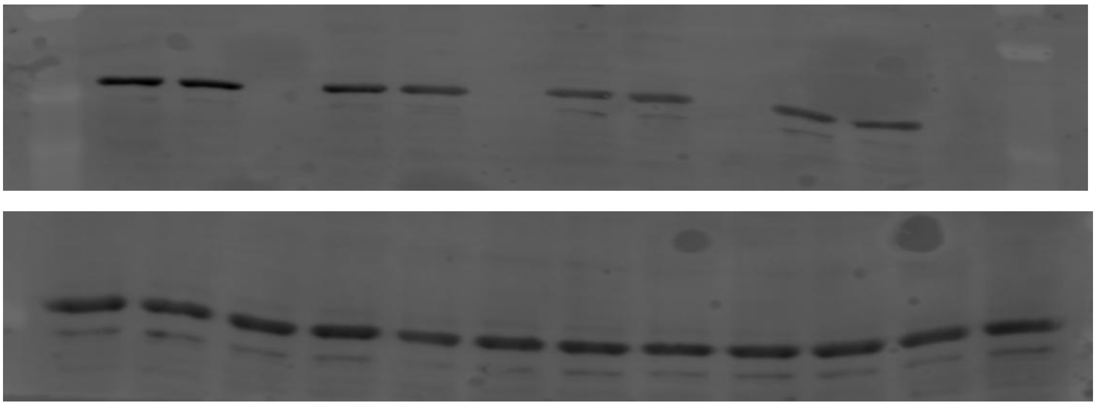

2. COPB2 knockdown suppresses the proliferation of RKO and HCT116 cells.

RKO：celigo

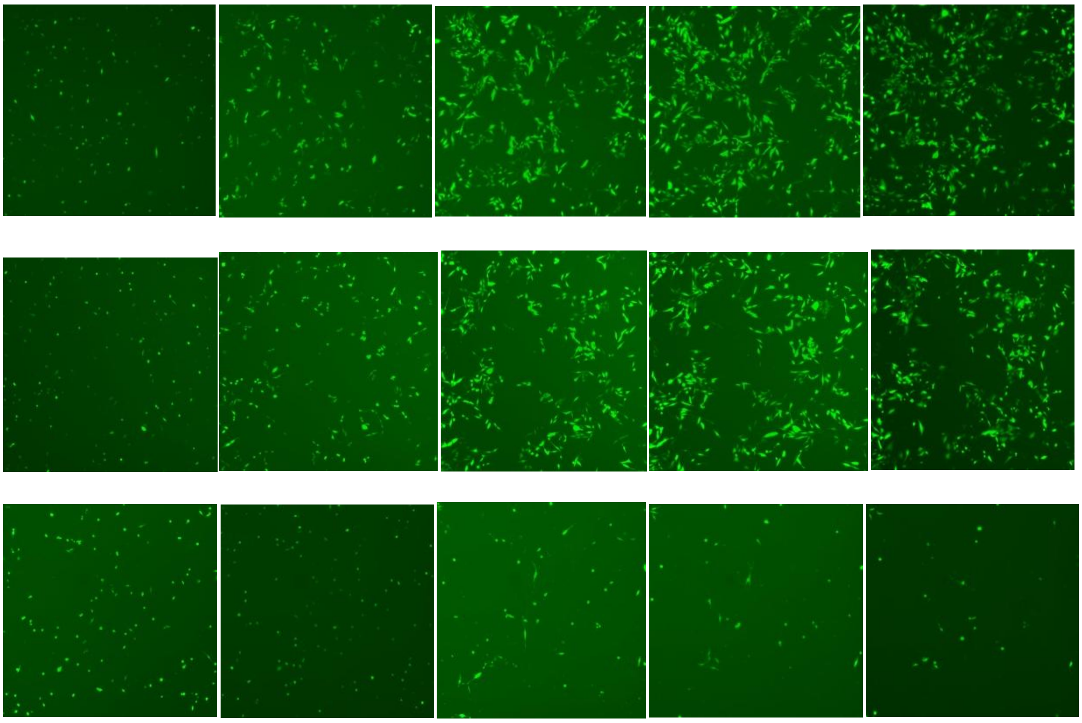

HCT116：celigo

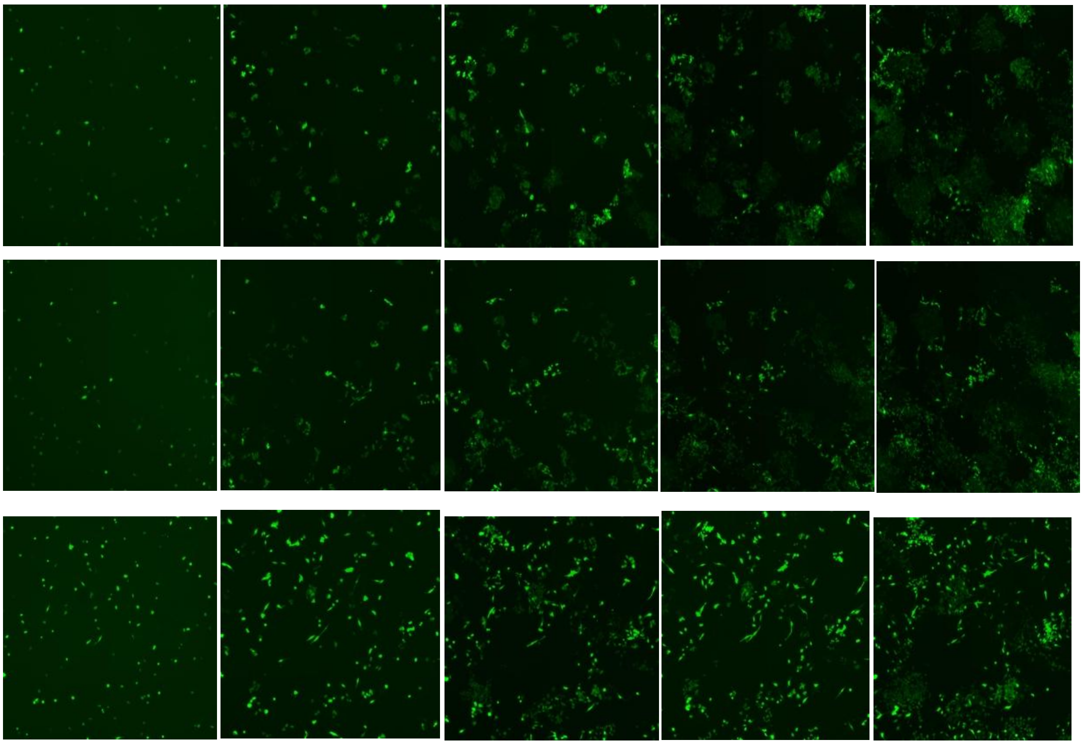

3. COPB2 knockdown promotes apoptosis of RKO and HCT116 cells and increases Caspase 3/7 activity.

RKO:

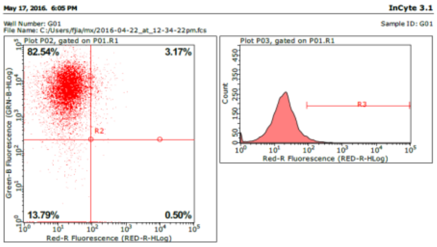
May 17, 2016, 6:05 PM
InCyte 3.1
Well Number: G01
Sample ID: G01
Plot P02, gated on P01.R1
82.54%
3.17%
13.79%
0.50%
R2
Green-B Fluorescence (GRN-B-HLog)
Red-R Fluorescence (RED-R-HLog)
Plot P03, gated on P01.R1
Count
R3
Red-R Fluorescence (RED-R-HLog)

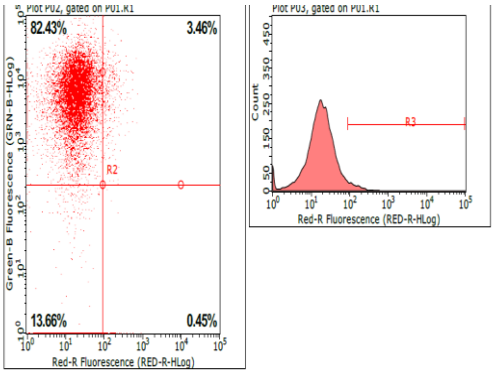
82.43%
3.46%
13.66%
0.45%
R2
Green-B Fluorescence (GRN-B-HLog)
Red-R Fluorescence (RED-R-HLog)
Count
R3
Red-R Fluorescence (RED-R-HLog)

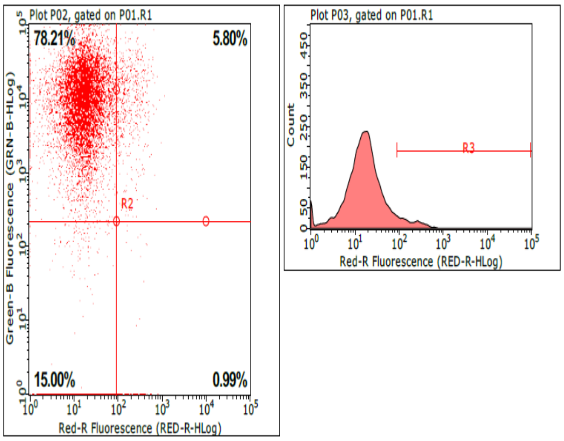
Plot P02, gated on P01.R1
78.21%
5.80%
15.00%
0.99%
R2
Green-B Fluorescence (GRN-B-HLog)
Red-R Fluorescence (RED-R-HLog)
Plot P03, gated on P01.R1
Count
R3
Red-R Fluorescence (RED-R-HLog)

HCT116:

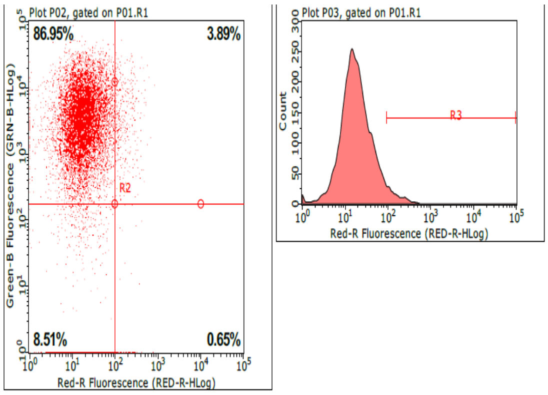
Plot P02, gated on P01.R1
86.95%
3.89%
8.51%
0.65%
R2
Green-B Fluorescence (GRN-B-HLog)
Red-R Fluorescence (RED-R-HLog)
Plot P03, gated on P01.R1
Count
R3
Red-R Fluorescence (RED-R-HLog)

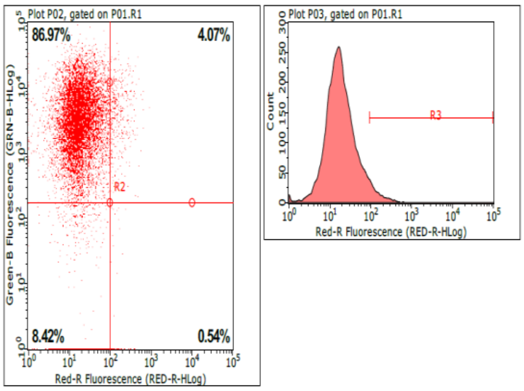
Plot P02, gated on P01.R1
86.97%
4.07%
8.42%
0.54%
R2
Green-B Fluorescence (GRN-B-HLog)
Red-R Fluorescence (RED-R-HLog)
Plot P03, gated on P01.R1
Count
R3
Red-R Fluorescence (RED-R-HLog)

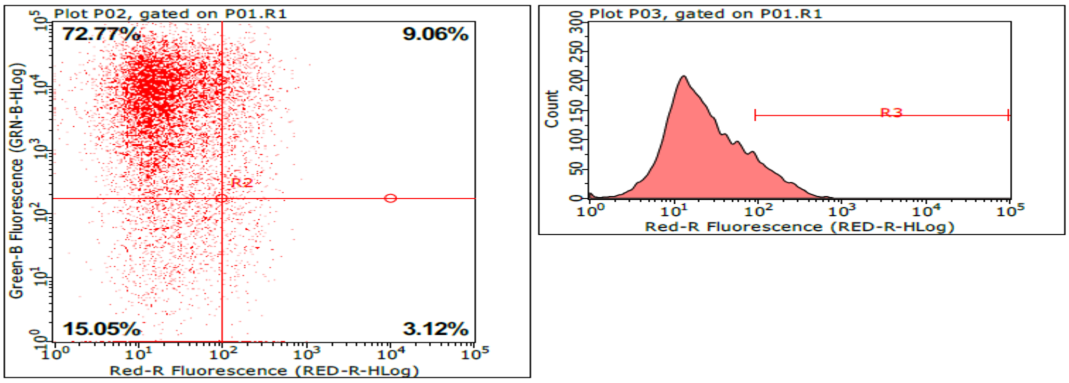
Plot P02, gated on P01.R1
72.77%
9.06%
15.05%
3.12%
R2
Green-B Fluorescence (GRN-B-HLog)
Red-R Fluorescence (RED-R-HLog)
Plot P03, gated on P01.R1
Count
R3
Red-R Fluorescence (RED-R-HLog)

4. Western blot analysis of apoptosis-related proteins expression in RKO and HCT116 cells.

Bcl-2:

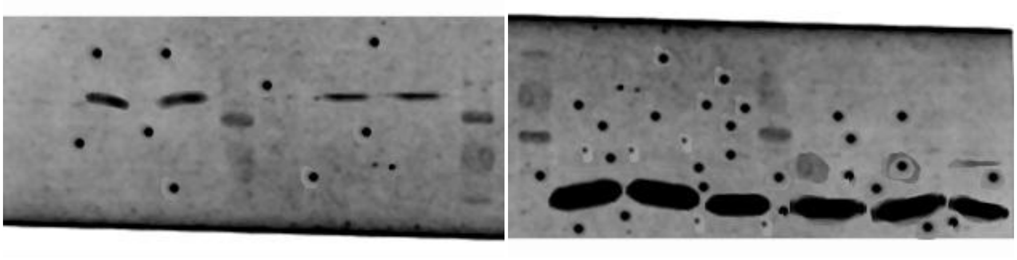

Bcl-2 GAPDH

Bax:

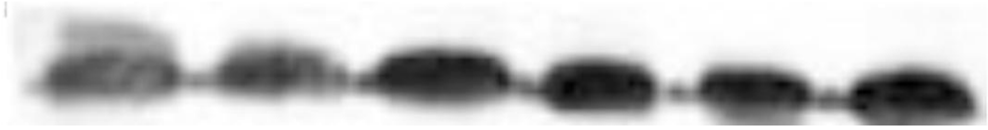

Bax

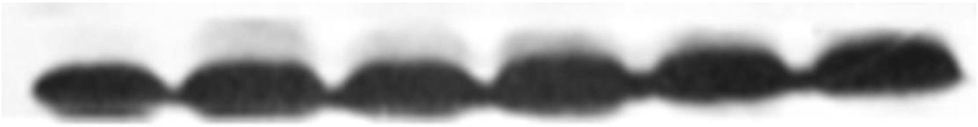

GAPDH

5. Western blot analysis of JNK and Jun protein expression in RKO and HCT116 cells.

RKO:

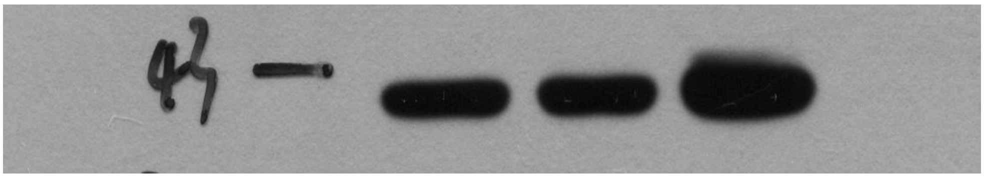


Jun

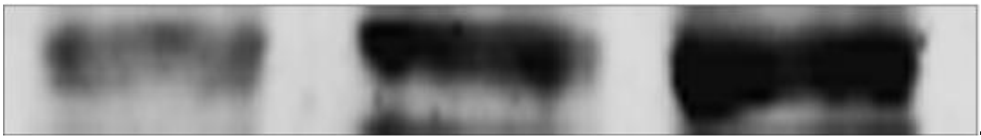

JNK

HCT116:

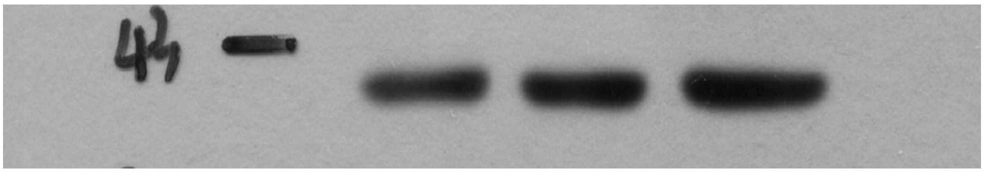


Jun

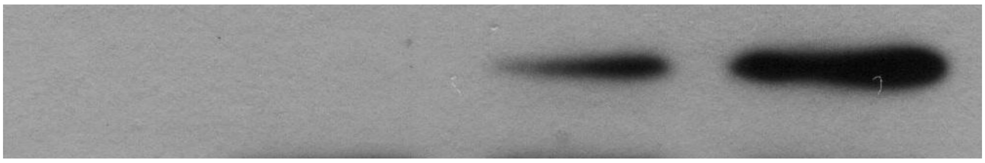

jnk

6. Western blot analysis of the expression levels of JNK/c-Jun signaling pathway related proteins in RKO and HCT116 cells.

RKO:

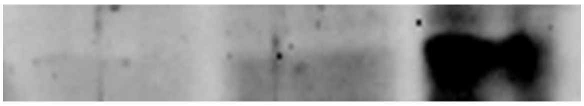

Bad

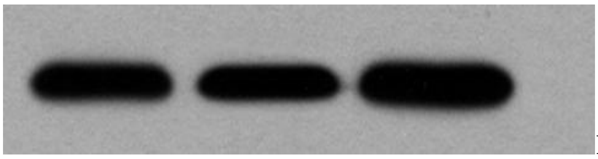

P53

HCT116:

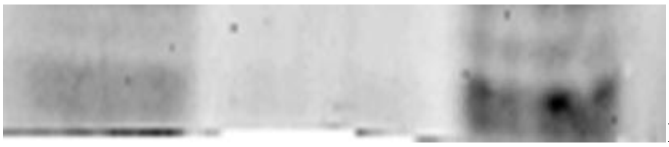

BAD

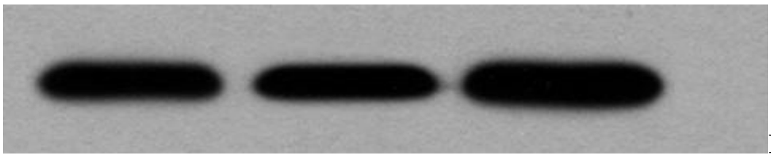

P53

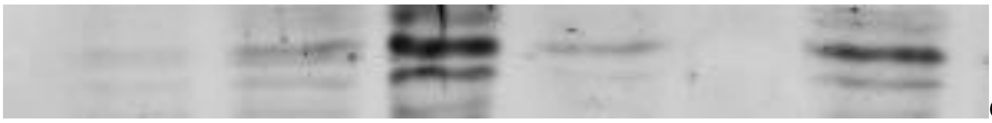

Caspase3

RKO HCT116

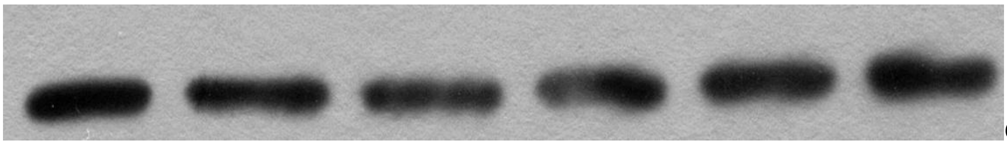

GAPDH
